# Pulmonary and Systemic Skeletal Muscle Embolism in a Beaked Whale with a Massive Trauma of Unknown Aetiology

**DOI:** 10.3390/ani12040508

**Published:** 2022-02-17

**Authors:** Eva Sierra, Tania Ramírez, Yara Bernaldo de Quirós, Marina Arregui, Blanca Mompeó, Miguel A. Rivero, Antonio Fernández

**Affiliations:** 1 Division of Histology and Animal Pathology, University Institute of Animal Health and Food Security (IUSA), Universidad de Las Palmas de Gran Canaria (ULPGC), 35413 Las Palmas, Canary Islands, Spain; eva.sierra@ulpgc.es (E.S.); tania.ramirez101@alu.ulpgc.es (T.R.); yara.bernaldo@ulpgc.es (Y.B.d.Q.); marina.arregui@ulpgc.es (M.A.); miguel.rivero@ulpgc.es (M.A.R.); 2 Department of Morphology, Universidad de Las Palmas de Gran Canaria (ULPGC), 35016 Las Palmas, Canary Islands, Spain; blanca.mompeo@ulpgc.es

**Keywords:** skeletal muscle, rete mirabile, embolism, beaked whales, pathology, cetaceans, traumatic event, rib fractures

## Abstract

**Simple Summary:**

A severe trauma of unknown aetiology was suspected as the cause of death in an adult female Sowerby’s beaked whale found floating dead in the Canary Islands in December 2016. Many bruises in the skin and muscles (contusions) were observed in the chest wall and bone fractures, mainly located in the mandible and ribs. The broken rib bones also affected thoracic muscles, which escaped into the blood circulation once ruptured, reaching several organic locations, including the lungs, where they became trapped within the small lumen of pulmonary blood vessels, leading to a systemic and pulmonary skeletal muscle embolism. An embolism occurs when a piece of intravascular internal or foreign material obstructs the lumen of a blood vessel, starving tissues of blood and oxygen. An embolism necessarily needs cardiac function, indicating a survival time after trauma. This case report aimed to include the diagnosis of skeletal muscle embolism as a routine tool to determine if the traumatic event occurred before or after death. This is especially valuable when working with dead animals because no other evidence of traumatic injury may be recorded if carcasses are in advanced decay.

**Abstract:**

An adult female Sowerby’s beaked whale was found floating dead in Hermigua (La Gomera, Canary Islands, Spain) on 7 December 2016. Severe traumas of unknown aetiology were attributed, and the gross and microscopic findings are consistent with catastrophic trauma as a cause of death. Rib fractures affected the intercostals, transverse thoracis skeletal muscles, and thoracic rete mirabile. Degenerated muscle fibres were extruded to flow into vascular and lymphatic vessels travelling to several anatomic locations into the thoracic cavity, including the lungs, where they occluded the small lumen of pulmonary microvasculature. A pulmonary and systemic skeletal muscle embolism was diagnosed, constituting the first description of this kind of embolism in an animal. The only previous description has been reported in a woman after peritoneal dialysis. Skeletal pulmonary embolism should be considered a valuable diagnostic for different types of trauma in vivo in wild animals. This is especially valuable when working with decomposed carcasses, as in those cases, it is not always feasible to assess other traumatic evidence.

## 1. Introduction

Several types of pulmonary embolism have been described in human and animal medicine, but generally, they can be grouped as thrombotic and non-thrombotic [1,2,3,4]. The latter is defined as the embolisation to the pulmonary vasculature of a wide range of non-thrombotic agents, including endogenous and exogenous material. Skin, fat, bone, bone marrow, amniotic fluid, trophoblast, decidua, brain, liver, fat, and bile are among the biological endogenous extravascular tissues previously reported to embolise the pulmonary vessels [5]. In addition, a single case of skeletal muscle pulmonary embolisation has been described in the literature, which occurred after peritoneal dialysis in a 75-year-old woman [6]. As in that case, most of these embolic tissue fragments usually happen due to a severe musculoskeletal and/or soft tissue trauma [7]. After trauma, cells from the damaged tissue enter the bloodstream through ruptured vessels (veins and venules) in the injury or fracture site, and they may reach the lung circulation, where they become trapped within the pulmonary microvasculature [8]. However, several mechanisms have been suggested for this process: emboli can be forced to enter the vessels or travel from an extravascular place inside the venous system due to a pressure gradient and/or a perforated vessel wall. The exact mechanisms of transvascular migration remain unclear [5,9].

Imaging diagnosis, such as computed tomography, can become difficult in cases of microemboli, in which the diagnosis is then based on parenchymal findings. A definitive diagnosis is reached by microscopic emboli visualisation, although indirect signs of obstruction include pulmonary oedema, infarction, empyema, or pulmonary hypertension [5,10]. The detection of endogenous biological origin embolism substances in a corpse indicates survival after trauma [7].

## 2. Materials and Methods

**Figure 1:**
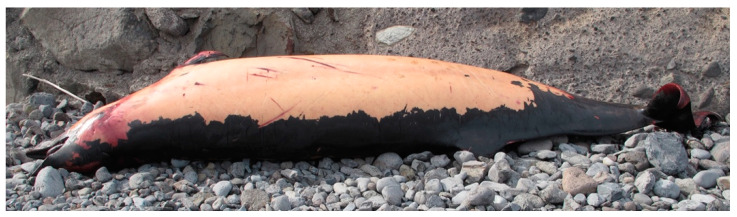
Ventro-lateral view of the stranded Sowerby’s beaked whale placed on the rocky coast where a standardised complete necropsy was performed. Large areas of epidermal sloughing were appreciable on the right side.

An adult female Sowerby’s beaked whale (*Mesoplodon bidens*) (total body length of 470 cm) was found floating dead in Hermigua (La Gomera, Canary Islands, Spain) on 7 December 2016 (CET 827). Presumably, this same animal had been previously sighted alive, with a calf, close to a rocky coast. After floating in the sea for a few days, the animal was finally towed to shore, where a necropsy was performed according to standard procedures [11,12,13] (Figure 1).

Biological parameters (stranding epidemiology (type, location, and date), life history data (species, age category, and sex)), body condition, and decomposition code were recorded. Age categories (neonate, calf, juvenile, and adult) were based on total body length and gonadal development [11,12,13]. Body condition (very poor, poor, fair, or good) was estimated based on anatomical landmarks [14]. Five codes of conservation condition were established: Code 1 (extremely fresh carcass, as an animal that has recently died or been euthanised), Code 2 (fresh carcass), Code 3 (moderate decomposition), Code 4 (advanced decomposition), and Code 5 (mummified or skeletal remains) [11,12,13]. During the necropsy, lesions were described and photographed.

### 2.1. Evidence of Ethical Approval

Permission for the management of stranded cetaceans was issued by the environmental department of the Canary Islands Government and the Spanish Ministry of Environment.

### 2.2. Gross, Histological and Histochemical Analyses

During necropsy, formalin-fixed samples for histopathologic processing were collected from selected tissues. The fixed tissue samples were then trimmed, routinely processed, embedded in paraffin, sectioned at 5 μm, and stained with haematoxylin and eosin (HE) for examination by light microscopy. Additionally, several tissue sections were cut at 4 μm for Phosphotungstic Acid Hematoxylin (PTAH) staining, as previously described [15]. PTAH is ideal for demonstrating striated muscle fibres, stained in blue, in contrast to collagen, stained in red.

### 2.3. Immunohistochemical Analyses

To confirm the presence of skeletal muscle fibres inside blood vessels, immunohistochemical stain with α-actin (HHF35; mouse anti-human monoclonal; Enzo Biochemical, New York, NY, USA) was employed. Briefly, 3 µm-thick sections were deparaffinised and rehydrated through a series of graded alcohols. Endogenous peroxidase activity was blocked by incubation in 3.0% hydrogen peroxide (30%) in methanol for 30 min. Protease digestion at 0.01% for 10 min was used for antigen retrieval. The sections were blocked with 10% normal rabbit serum in phosphate-buffered saline (PBS) for 30 min. The primary antibody (1/200 dilution) was incubated overnight at 4 °C. The sections were washed in PBS and incubated for 30 min with a 1:20 biotinylated rabbit anti-mouse secondary antibody. The label used was an avidin–biotin–peroxidase complex (Elite ABC kit, Vector Laboratories, Burlingame, CA, USA), following the manufacturer’s instructions. 3-Amino-9-ethyl-carbazole (AEC, Sigma Chemical Co., St. Louis, MO, USA) was used as the chromogen. Slides were pre-incubated in acetate buffer for 10 min and incubated for 1 min with a filtered solution of AEC (0.025 g AEC was dissolved in 5 mL N, N-dimethylformamide, 70 mL acetate buffer, and 75 µL hydrogen peroxide at 30%). Sections were rinsed in tap water, counterstained with Mayer’s haematoxylin, and mounted with an aqueous medium. Nonimmune homologous serum was used as the negative control. Anti-muscle-specific actin recognises alpha and gamma isotypes of all muscle groups. Cross-reactivity in cetacean skeletal muscle has been previously demonstrated [16]. Anti-von Willebrand factor (RRID: AB_2811207, rabbit polyclonal; Thermo Fisher Scientific) antibody was used as previously described [17] and visualised using a Chemmate Dako EnVision detection kit (Glostrup—Denmark).

### 2.4. Molecular Virological Analyses

Molecular analysis for screening of herpesvirus and cetacean morbillivirus (CeMV) were performed in selected frozen tissues (i.e., skin, skeletal muscle, lung, liver, mesenteric lymph node, kidney, brain, and spleen). Herpesvirus DNA was detected by conventional nested PCR using degenerate primers designed to amplify a region of the DNA polymerase gene [18]. Molecular detection of cetacean morbillivirus was performed by a one-step reverse transcription-quantitative PAN RT-qPCR method based on SYBRN^®^ Green dye that successfully detects different CeMV strains and amplifies a 205 bp partial region of the P gene [19].

## 3. Results

### 3.1. Gross Findings

**Figure 2:**
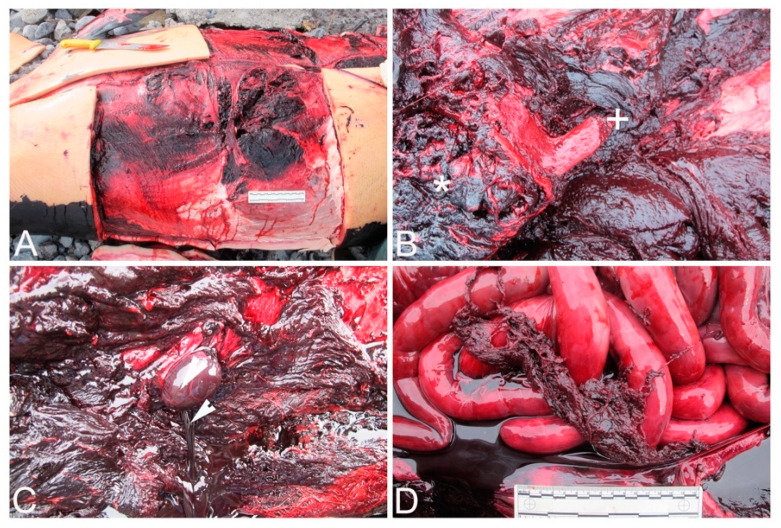
Gross findings. (**A**). A perforated wound in the right lateral thoracic region. (**B**). Multifocal extremely friable skeletal muscle (asterisk) in areas of contusion and rib fractures (plus sign). (**C**). Haemoabdomen. Abundant serosanguinolent fluid flowing from the abdominal cavity (arrowhead). (**D**). Several clots adhered to the intestine, confirming the presence of blood in the peritoneum.

The postmortem examination revealed several haematomas in the dorsal and ventral aspects of the head and thorax (Figure 2A), affecting the hypodermis, the fascia, and the muscular tissues. The skeletal muscles (epaxial and hypaxial skeletal musculature) were extremely friable, particularly close to their attachment to the thoracic vertebrae (Figure 2B). In addition, locally extensive haemorrhages in epaxial muscles near costochondral joints and within the thoracic wall (affecting the intercostal muscles) were observed. Multiples fractures were found in the right ribs (Figure 2B), which were simple in nature with a slight degree of external bone displacement into adjoining soft tissues. Simple fractures with bone displacement were also observed in both mandibles. A moderate haemoabdomen and systemic vascular changes (congestion, haemorrhages, and clots) were also present (Figure 2C,D). The lungs were collapsed with subpleural emphysema, most remarkably in the right upper lobe.

### 3.2. Histopathological and Histochemical Findings

**Figure 3:**
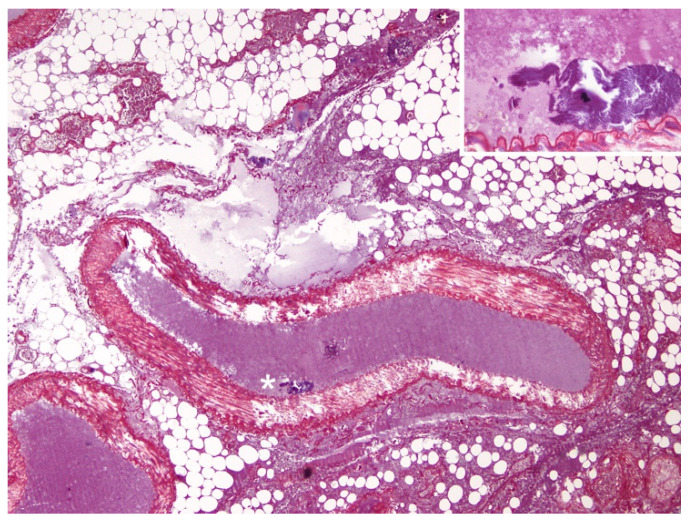
Rete mirabile, microscopic findings. Presence of ruptured myofibers (PTAH blue-stained) inside an artery (asterisk) (red PTAH-stained collagen is visible in the wall artery). PTAH, 10×. Inset: high magnification of the skeletal muscle cells inside the artery.

**Figure 4:**
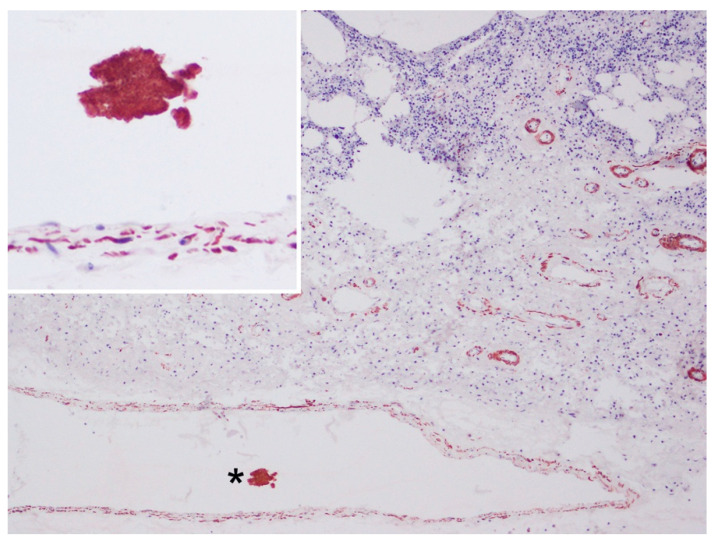
Pulmonary lymph node, microscopic findings. Presence of a fragmented myofiber (asterisk) (strong immunostained with α-actin) inside an artery (smooth muscle cells of vessel walls were also α-actin positive). Immunohistochemistry for α-actin, 10×. Inset: high magnification of the skeletal muscle cells inside the artery.

**Figure 5:**
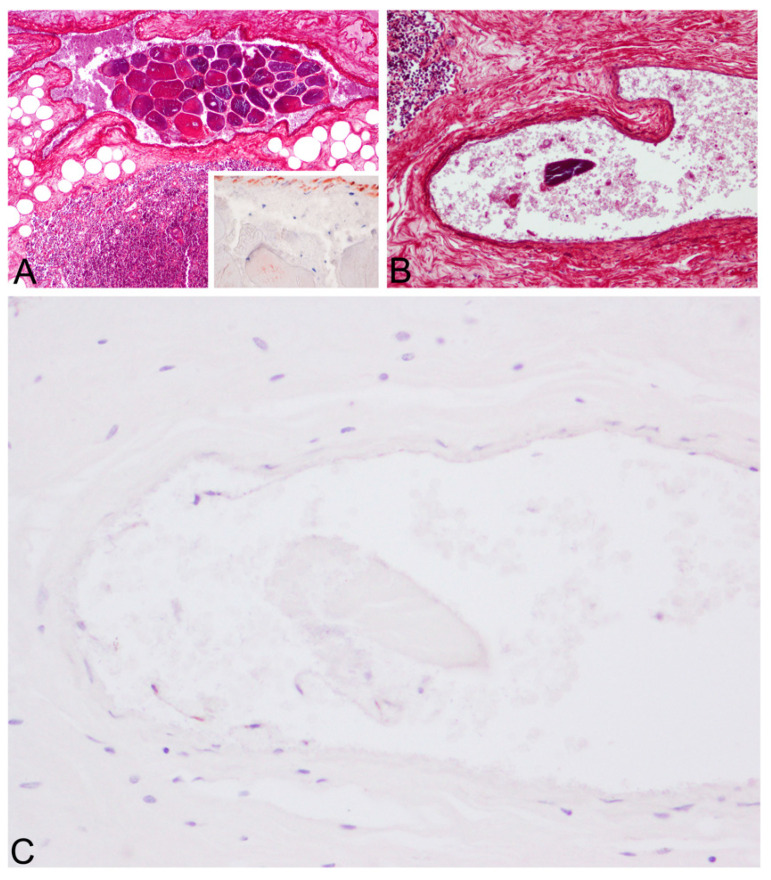
Thymus capsule, microscopic findings. (**A**). Thymus capsule. Embolus consisting of a bundle of skeletal muscle fibres (blue and red (degenerated)-PTAH stained). PTAH. 10×. Inset: weak α-actin immunoreactivity in degenerated skeletal muscle fibres inside an artery (arrowhead) (strong α-actin immunolabeling of smooth muscle of the arterial wall). (**B**). Presence of a fragmented myofiber (blue-PTAH stained) inside an artery (arrowhead). PTAH, 20×. (**C**). Inset: confirmation of skeletal muscle embolisation by anti-von Willebrand factor immunolabeling the endothelial cells (arrowheads) (most of them are desquamated). Anti-von Willebrand factor antibody, 40×.

**Figure 6:**
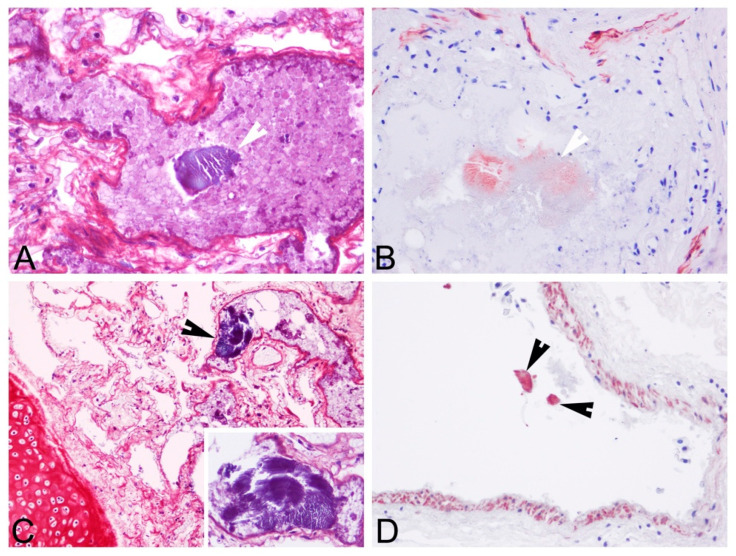
Lung microscopic findings. (**A**). Embolisation of a ruptured myofiber (blue stained with PTAH stain; arrowhead). HE, 20×. (**B**). Moderate immunolabeling of an embolised skeletal muscle fibre (arrowhead) (strong α-actin immunolabeling of smooth muscle of the arterial wall). α-actin immunohistochemistry, 20×. (**C**). Embolisation of a ruptured myofiber (blue-stained with PTAH stain; arrowhead). HE, 10×. Inset: high magnification of the embolised skeletal muscle fibre with the characteristic striation pattern). (**D**). Strong immunolabeling of embolised skeletal muscle cells (arrowheads) (strong α-actin immunolabeling of smooth muscle of the arterial wall). α-actin immunohistochemistry, 20×.

Upon histopathologic and histochemical examinations, the main findings were moderate to severe skeletal muscle segmentary hypercontraction and myonecrosis with the presence of intravascular myofibers in different locations, specifically in the rete mirabile (Figure 3), subcapsular sinus in the prescapular lymph node, lymphoid capsule in pulmonary lymph node (Figure 4), thymus capsule (Figure 5) and lung (Figure 6). Severe haemorrhages were found in the prescapular lymph node and rete mirabile. In the latter, these haemorrhages were associated with disrupted tissue intermixed with free adipose tissue and necrotic muscle fibres, as well as vessels wall damage. Myofibers showed blue staining by PTAH, turning to red-purple when disrupted (degenerated), confirming myofibers both free in the rete mirabile and inside blood vessels.

### 3.3. Immunohistochemical Findings

Myofibers were also identified by α-actin antibody displaying different levels of immunostaining (weak/moderate/strong) (Figure 4, Figure 5 and Figure 6). The reduction in immunostaining intensity observed in some of the embolised skeletal muscle fibres could be due to the loss of skeletal muscle integrity (degeneration). As expected, α-actin immunolabeling was also observed in the smooth muscle cells of vessel walls. In addition to the anti-von Willebrand factor antibody (Figure 5C), this immunolabeling substantiates that muscle tissue was placed in a vessel.

### 3.4. Molecular Results

All the tested samples were negative to HV [20] and CeMV (under review).

## 4. Discussion

Severe pulmonary and systemic gas and fat embolism, compatible with decompression sickness (DCS), has been described in beaked whales related to mid-frequency active sonar during military manoeuvres [21]. In those cases, gas bubble-associated lesions and fat embolisms were observed in the vessels and parenchyma of vital organs. A modified diving behaviour and/or physiological stress reaction overriding the diving response in response to sonar exposure has been postulated as an explanation to trigger gas bubble formation in those animals. Nitrogen can dissolve easily into the bloodstream and tissues under high pressure at great depths. Still, it can come out of solution when the sum of partial pressures exceeds the environmental pressure, forming gas bubbles in the blood (gas emboli) and body tissues, causing local and systemic damage [22]. In contrast, pulmonary and systemic fat emboli are usually the consequence of direct injury (trauma) to fat depots [23,24,25], as has also been proved for sperm whales that have died due to vessel collisions [26].

Beaked whales are extreme deep divers, a condition that renders them prone to a higher susceptibility to suffering from DCS-like syndromes [22]. Here, we present a pulmonary and systemic muscle embolism in a Sowerby’s beaked whale. Gross and microscopic lesions were consistent with massive trauma, which would have been sufficiently severe to account for the death of this animal. The nature of the trauma in the Sowerby’s beaked whale of our study remains undetermined. Still, it should have been strong enough to cause several right ribs fractures and affect their associated thoracic muscles (intercostals and transverse thoracis origin and insert on the ribs) [27] and the rete mirabile. The rete mirabile is a complex structure in which an artery branches into multiple smaller vessels that eventually reconstitute one (or a few) larger vessels, thus creating a direct continuation of the artery that generated the rete [8,28]. The cervicothoracic rete mirabile is supplied by the brachiocephalic trunk and the intercostal and internal thoracic arteries [29]. It continues with the cervical rete mirabile, which enters the occipital foramen with the spinal meningeal arteries for cerebral blood circulation supply. The cervico-thoracic rete in marine mammals has afferent and efferent limbs (ambiocentric type) [28].

The force of the traumatic impact in the right thoracic wall of the Sowerby’s beaked whale of our study presumably forced muscular and fat fragments into the vessel channels. Skeletal muscles could arise from the thoracic musculature, while fat (not histochemically demonstrated in our study) could be released at fracture sites or may be derived from adipocyte-rich soft tissue injuries [8,30], as it is present in the histologically normal stroma of the rete mirabile [28,29]. These fragments are then likely to have been transported by the blood and lymphatic circulation, during the agonal period of survival, towards several anatomic locations, including the lungs, where they occluded the small lumen of pulmonary blood vessels [31]. The lung blood vessels receive the venous return from all the peripheral organs; thus, emboli arising in any part of the systemic circulation necessarily pass through the lungs [32]. Embolisation occurs most commonly in the lungs, and for that reason, the lung is considered the target organ for emboli detection. The thoracic scan is the method of choice in cases of suspected embolism [33].

The thorax has been described as the most affected body region with fractures involving the ribs in a retrospective study of traumatic intra-interspecific interactions in stranded cetaceans in the Canary Islands [34]. However, in cases of advanced autolysis carcasses, it is challenging to distinguish if the trauma occurred ante- or post-mortem. The presence of muscle and fat emboli within the lungs in our case constitutes evidence of antemortem injury, as cardiac function is needed, even for a short period, to disseminate these droplets to reach the lung circulation [35].

## 5. Conclusions

Skeletal pulmonary embolism should be considered a useful diagnostic criterion for different types of trauma in vivo in wild animals. This is especially valuable when working with decomposed carcasses, as assessing other traumatic evidence is not always feasible. This case is, to our knowledge, the first description of skeletal muscle embolism in an animal. The only previous description was reported in a woman.

## Data Availability

Data are contained within the article.
